# Membrane Proteomics to Understand Enhancement Effects of Millimeter-Wave Irradiation on Wheat Root under Flooding Stress

**DOI:** 10.3390/ijms24109014

**Published:** 2023-05-19

**Authors:** Setsuko Komatsu, Kazuna Hamada, Takashi Furuya, Takumi Nishiuchi, Masahiko Tani

**Affiliations:** 1 Faculty of Environment and Information Sciences, Fukui University of Technology, Fukui 910-8505, Japan; kazu920baske@gmail.com; 2 Research Center for Development of Far-Infrared Region, University of Fukui, Fukui 910-8507, Japan; furuya@fir.u-fukui.ac.jp (T.F.); tani@fir.u-fukui.ac.jp (M.T.); 3 Institute for Gene Research, Kanazawa University, Kanazawa 920-8640, Japan; tnish9@staff.kanazawa-u.ac.jp

**Keywords:** proteomics, wheat, membrane, millimeter-wave irradiation

## Abstract

Millimeter-wave irradiation of wheat seeds enhances the growth of roots under flooding stress, but its mechanism is not clearly understood. To understand the role of millimeter-wave irradiation on root-growth enhancement, membrane proteomics was performed. Membrane fractions purified from wheat roots were evaluated for purity. H^+^-ATPase and calnexin, which are protein markers for membrane-purification efficiency, were enriched in a membrane fraction. A principal-component analysis of the proteomic results indicated that the millimeter-wave irradiation of seeds affects membrane proteins in grown roots. Proteins identified using proteomic analysis were confirmed using immunoblot or polymerase chain reaction analyses. The abundance of cellulose synthetase, which is a plasma-membrane protein, decreased under flooding stress; however, it increased with millimeter-wave irradiation. On the other hand, the abundance of calnexin and V-ATPase, which are proteins in the endoplasmic reticulum and vacuolar, increased under flooding stress; however, it decreased with millimeter-wave irradiation. Furthermore, *NADH dehydrogenase*, which is found in mitochondria membranes, was upregulated due to flooding stress but downregulated following millimeter-wave irradiation even under flooding stress. The ATP content showed a similar trend toward change in *NADH dehydrogenase* expression. These results suggest that millimeter-wave irradiation improves the root growth of wheat via the transitions of proteins in the plasma membrane, endoplasmic reticulum, vacuolar, and mitochondria.

## 1. Introduction

Based on their frequency or wavelength, electromagnetic waves are typically divided into eight spectral bands: audio waves, radio waves, microwaves, infrared, visible light, ultraviolet, X-rays, and gamma rays [1]. Although electromagnetic energy is the backbone of wireless communication systems, its continuous use impacts a wide range of biological systems. The effects of electromagnetic waves on living organisms, which largely rely on the frequency and penetration depth of the waves in the medium [1], can either be positive or negative. On the positive side, electromagnetic waves are being used in agriculture for the protection of plants, the development of crop varieties, the management of insect pests, the monitoring of fertilizer efficiency, and the preservation of agricultural produce [2]. Despite these current utilizations and other promising applications, the effects of the absorption of electromagnetic radiation in plants are insufficiently addressed [2]. Since electromagnetic wave irradiation has been used for remarkable advancements in the field of agriculture, research should be carried out to further shed light on issues not yet addressed in this field.

Studying the different bands in the electromagnetic spectrum, microwave irradiation has the most effects on seed germination and plant growth. Low-intensity microwaves do not affect plant growth, but the increased irradiation of microwave slowed down the seed germination of Chinese cabbage [3]. In another study, microwave-irradiated *Brassica napus* showed higher seed germination, plant growth, biomass, photosynthetic pigments, and antioxidant-enzyme activity compared with untreated plants, which showed higher reactive-oxygen species and electrolyte leakage [4]. Moreover, the germination rate of Sorghum seeds increased in a relatively short irradiation period, indicating that plants positively responded to microwaves even after only a short period of irradiation [5]. In addition, it was revealed that treated plants were more tolerant of stresses caused by heavy metals than unirradiated plants [5]. It was also found that a suitable dose of microwave radiation can enhance the capability of wheat seedlings to eliminate free radicals induced by osmotic stress, resulting in an increase in resistance to osmotic stress [6]. All these findings indicate that a suitable dose of microwave radiation can increase tolerance against various environmental stresses.

Another useful type of radiation for agricultural applications is the millimeter wave (MMW), which lies between the microwave and infrared regions of the electromagnetic spectrum. This type of radiation opens up a broad horizon to wireless communication technologies, offering a broad bandwidth operating at the spectrum of 30 to 300 GHz [7]. The wavelength of the MMW ranges from 1 to 10 mm, which falls under the category of extremely high frequency, as per the International Telecommunication Union standards. However, despite its extensive applications, specially in the telecommunication sector, MMW can be easily affected by atmospheric attenuation due to molecular absorption by water vapor and harsh weather such as rain [8] and by propagation distance [9,10].

The non-thermal effects of MMW irradiation targeting crop seeds were studied using brown rice and wheat, showing that MMW irradiation improved germination in brown rice [11] and in wheat [12,13], as well as the subsequent growth of shoots and grains [14,15]. Another study showed that MMW irradiation affected proteins in grown soybean [16], chickpea [17], and wheat [18], the seeds of which were irradiated, under flooding stress. The MMW-irradiated seeds of soybean, chickpea, and wheat had shown improvement in the growth of plants and had displayed tolerance even under flooding stress. These previous results indicated that irradiation with MMW might be a feasible approach for the development of stress-tolerant lines, which will benefit the yield of the crop. The abovementioned morphophysiological effects of MMW irradiation on crops served as inspiration to conduct investigations on applications for increasing crop productivity in the agriculture industry. However, the mechanism behind this improvement is not yet clearly understood. To understand the mechanism of flooding stress tolerance in wheat irradiated with MMW, membrane proteomics is performed in this study. After obtaining the membrane fraction from wheat root, the membrane fractions are evaluated for purity and analyzed using proteomic technique. For a more clear understanding of the mechanism behind flooding stress tolerance, proteins identified using proteomic analysis are confirmed using immunoblot or polymerase chain reaction (PCR) analyses in this study.

## 2. Results

### 2.1. Membrane Purification and Purity Check of Wheat Root

To investigate the subcellular function in wheat root irradiated with MMW, membrane proteomics was performed. Before analyzing the effects of MMW irradiation, a membrane purification protocol was established. The roots of 5-day-old wheat were collected and purified for the membrane fraction. Proteins extracted from the membrane fraction were evaluated using an immunoblot analysis (Table S1). The staining pattern of Coomassie brilliant blue was used as a loading control (Figure S1). In the immunoblot analysis, anti-H^+^-ATPase and calnexin antibodies were used as protein markers of the plasma and endoplasmic-reticulum membranes, respectively. Furthermore, to assess protein contamination from the cytosolic fraction, purified membrane protein fractions were examined using the anti-cytosolic ascorbate peroxidase antibody.

**Figure 1:**
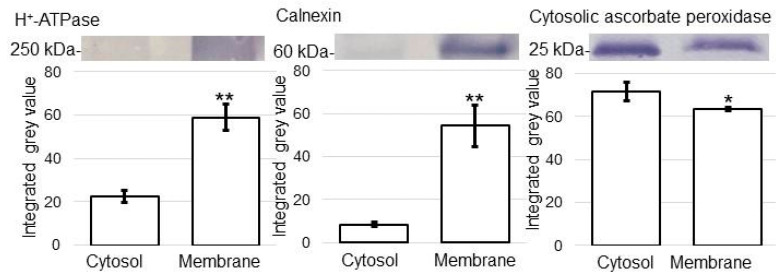
Purity evaluation of membrane fraction from wheat root. Proteins extracted from membrane fractions were evaluated using immunoblot analysis. The staining pattern of Coomassie brilliant blue was used as a loading control (Figure S1). In the immunoblot analysis, anti-H^+^-ATPase, calnexin, and ascorbate peroxidase antibodies were used as protein markers of plasma, endoplasmic reticulum membranes, and cytosol, respectively. Experiments were performed with three independent biological replicates (Figure S2). Data are shown as means ± SD from three biological replicates. Student’s *t*-test was used to compare values between control and treatment. Asterisk indicates a significant change (* *p* ≤ 0.05, ** *p* ≤ 0.01).

H^+^-ATPase and calnexin were significantly accumulated in the membrane fraction compared with in the cytosolic fraction (Figure S2 and Figure 1). Cytosolic ascorbate peroxidase was used as the protein marker of the cytosol. The cytosolic fraction experienced more accumulation of cytosolic ascorbate peroxidase compared with the membrane fraction (Figure 1). On the basis of the immunoblot analysis of subcellular-specific proteins, the membrane fraction was confirmed to be highly enriched with membrane proteins and was not contaminated much with proteins from the cytosol. This purification protocol for the membrane fraction was used to investigate the subcellular function in wheat root irradiated with MMW.

### 2.2. Membrane Proteomics of Wheat Irradiated with MMW

**Figure 2:**
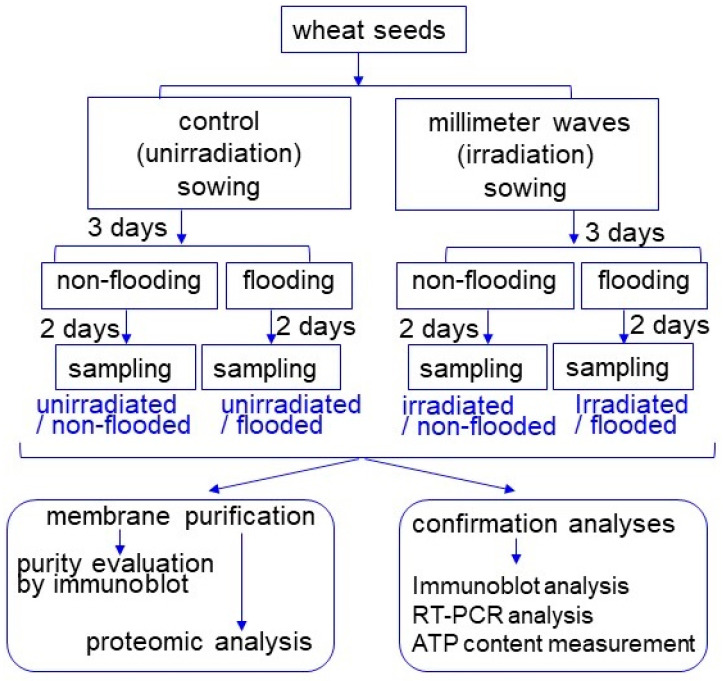
Experimental design of membrane purification, membrane proteomics, and confirmation analyses used in this study. Wheat seeds were irradiated with or without MMW. Three-day-old wheat was treated with or without flooding stress for 2 days. Membrane fraction was purified from root, evaluated for purity, and analyzed using proteomic technique. Proteomic results were confirmed via immunoblot, RT-PCR, or ATP-content analyses. All experiments were performed with three independent biological replicates.

To identify membrane proteins affected by MMW irradiation in wheat, a gel-free/label-free proteomic technique was used. Seeds were irradiated with or without 20 mW of MMW for 20 min. Seeds with or without MMW irradiation were sown, and the 3-day-old seedlings were treated with flooding stress for 2 days (Figure 2). Membrane fractions were extracted from the roots of 5-day-old seedlings, and the extracted membrane proteins were digested and analyzed using nano-liquid chromatography (LC)–mass spectrometry (MS)/MS. The identified proteins of wheat irradiated with MMW were compared with those of unirradiated wheat using PERSEUS software.

**Figure 3:**
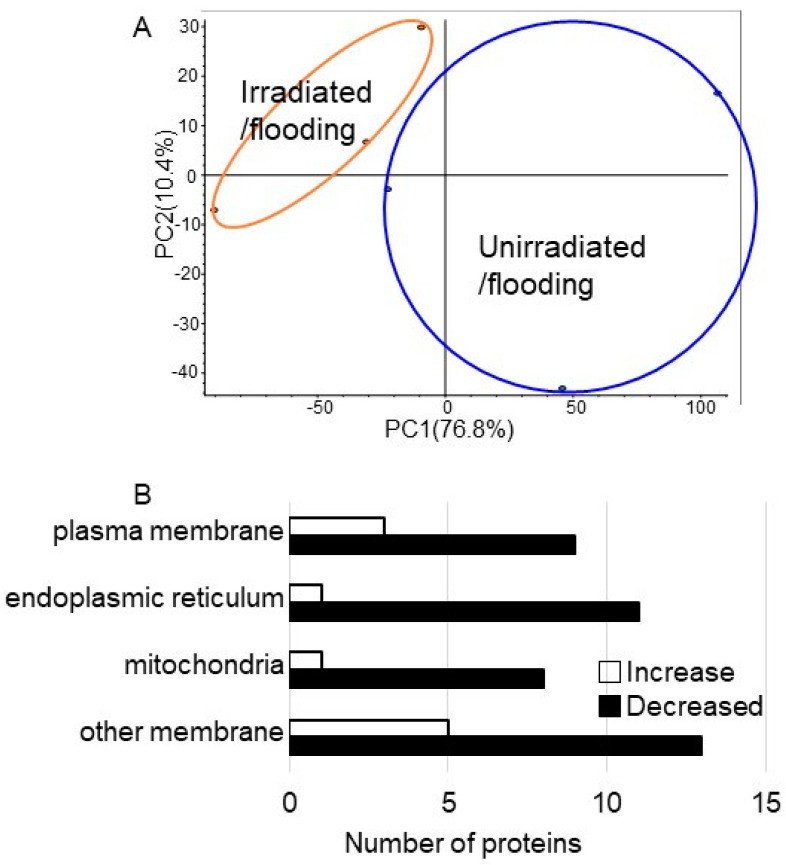
Overview of total proteomic data and cellular localization of membrane proteins in wheat irradiated with MMW under flooding stress. Wheat seeds were irradiated with or without MMW and 3-day-old wheat treated with flooding stress for 2 days. (**A**) Overview of total proteomic data (Table S2) from six samples of membrane fractions of wheat based on principal component analysis. Proteomic analysis was performed with three independent biological replicates for irradiated/flooding (orange color) and unirradiated/flooding (blue color). Principal component analysis was performed with Proteome Discoverer 2.2. (**B**) The cellular localization of membrane proteins with differential abundance in membrane fraction in wheat. After proteomic analysis, proteins that significantly changed (Table 1) in wheat membrane fraction were compared for the cases with and without MMW irradiation. The cellular localization of proteins that changed were determined using gene-ontology analysis. White and black columns show the increase and decrease in the amount of proteins, respectively, in MMW-irradiated wheat compared with unirradiated wheat under flooding stress.

The proteomic data were estimated using a principal component analysis, which indicated the difference between the control and treated samples (Figure 3A). Proteins with more than two matched peptides and less than 0.05 *p*-values were identified, and 246 proteins were determined in the membrane fraction in wheat root (Table S2). To better understand the function of the identified proteins, the differentially accumulated proteins were analyzed using the gene-ontology database. As significantly changed proteins, the amounts of 10 and 41 proteins increased and decreased, respectively, with irradiation compared with without irradiation under flooding stress (Figure 3B, Table 1). In the changed membrane proteins, proteins for which their amounts significantly increased and decreased were further confirmed using immunoblot or PCR analyses.

### 2.3. Protein Abundance in Plasma Membrane, Vacuole Membrane, and Endoplasmic Reticulum Membrane Modified with MMW Irradiation under Flooding Stress

**Figure 4:**
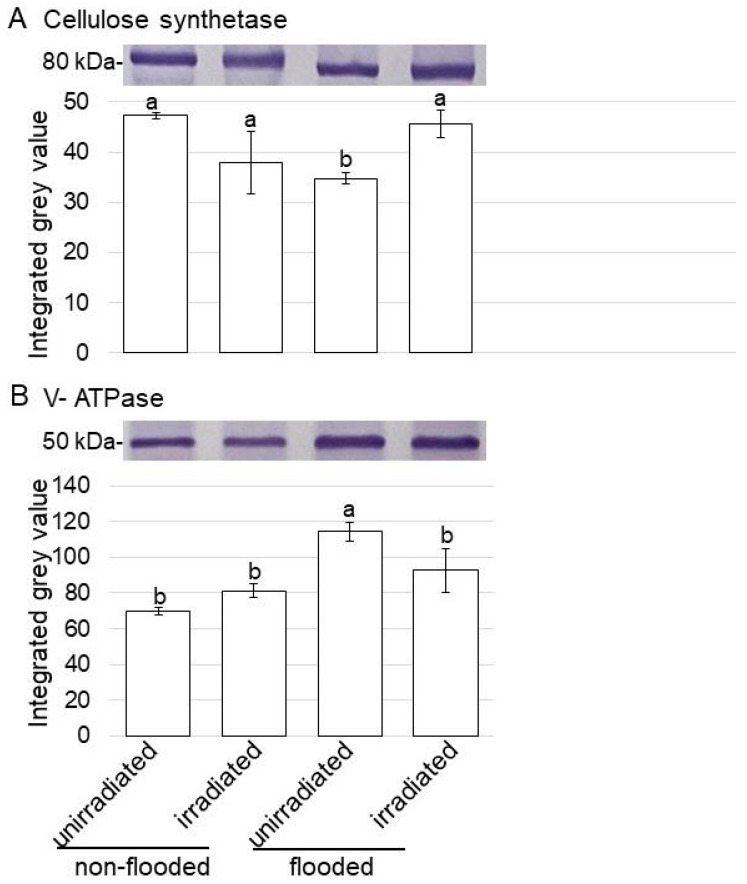
Immunoblot analysis of cellulose synthetase and V-ATPase in wheat irradiated with MMW under flooding stress. Proteins extracted from the roots of seedlings were separated on SDS-polyacrylamide gel and transferred onto membranes. The membranes were cross-reacted with anti-cellulose synthetase (**A**) and anti-V-ATPase (**B**) antibodies. Staining pattern with Coomassie brilliant blue was used as a loading control (Figure S3). The integrated densities of bands were calculated using ImageJ software. The data are given as the mean ± SD from biological triplicates (Figures S4 and S5). Mean values in each point with different letters are significantly different according to one-way ANOVA followed by Tukey’s multiple comparisons (*p* ≤ 0.05).

**Figure 5:**
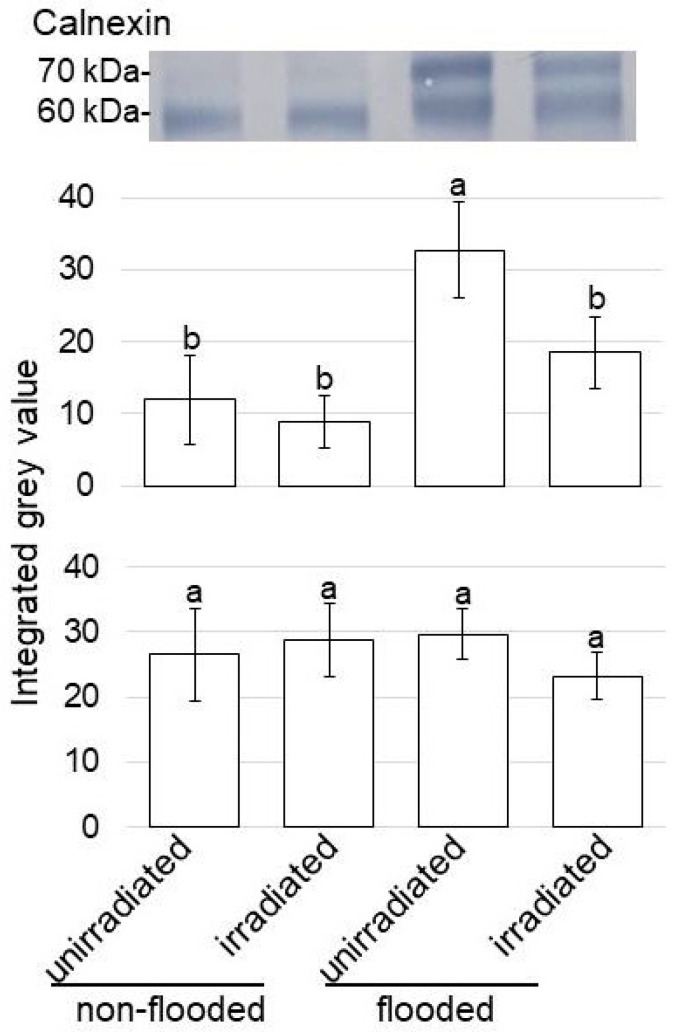
Immunoblot analysis of calnexin in wheat irradiated with MMW under flooding stress. Proteins extracted from the roots of wheat seedlings were separated on SDS-polyacrylamide gel via electrophoresis and transferred onto membranes. The membranes were cross-reacted with anti- calnexin antibody. Staining pattern with Coomassie brilliant blue was used as a loading control (Figure S3). The integrated densities of bands were calculated using ImageJ software. The data are given as the mean ± SD from biological triplicates (Figure S6). Mean values at each point with different letters are significantly different according to one-way ANOVA followed by Tukey’s multiple comparisons (*p* ≤ 0.05).

To further reveal the changes in the accumulation of proteins from various treatments, an immunoblot analysis of protein change in the plasma membrane, the vacuole membrane, and the endoplasmic reticulum membrane was carried out. Proteins were extracted from the roots of wheat, the seeds of which were treated with or without 20 mW of MMW irradiation for 20 min under non-flooded or flooded conditions (Figure 2). The protein extracts were separated on SDS-PAGE, and the staining pattern of Coomassie brilliant blue was used as a loading control (Figure S3). Proteins separated on SDS-PAGE were transferred onto membranes and cross-reacted with the anti-cellulose synthase, anti-V-ATPase, and anti-calnexin antibodies (Figures S4–S6). The relative band intensities were calculated using Image J software (Figure 4 and Figure 5).

The amount of 80 kDa cellulose synthase in unirradiated wheat significantly decreased with flooding stress compared with that in the non-flooding condition (control); however, following this reduction, the amount recovered to control levels in irradiated wheat, even with flooding stress (Figure 4A). On the other hand, the amount of 50 kDa V-ATPase significantly increased with flooding stress compared with that in the control; however, following this accumulation, the amount recovered to control levels in irradiated wheat, even with flooding stress (Figure 4B). Although the amount of 60 kDa calnexin did not change with any treatments, the amount of 70 kDa calnexin significantly increased with flooding stress compared with that in the control (Figure 5). Furthermore, following this accumulation, the amount recovered to control levels in irradiated wheat, even with flooding stress (Figure 5).

### 2.4. Expression of Gene That Encoded Protein in Mitochondrial Membrane Modified with MMW Irradiation under Flooding Stress

Based on the proteomic results, the change in the expression level of the gene that encoded proteins in the mitochondrial membrane under flooding stress with MMW irradiation was confirmed with a PCR analysis. *NADH dehydrogenase*-specific oligonucleotides were used to amplify transcripts of the total RNA isolated from the wheat root (Figure 2).

**Figure 6:**
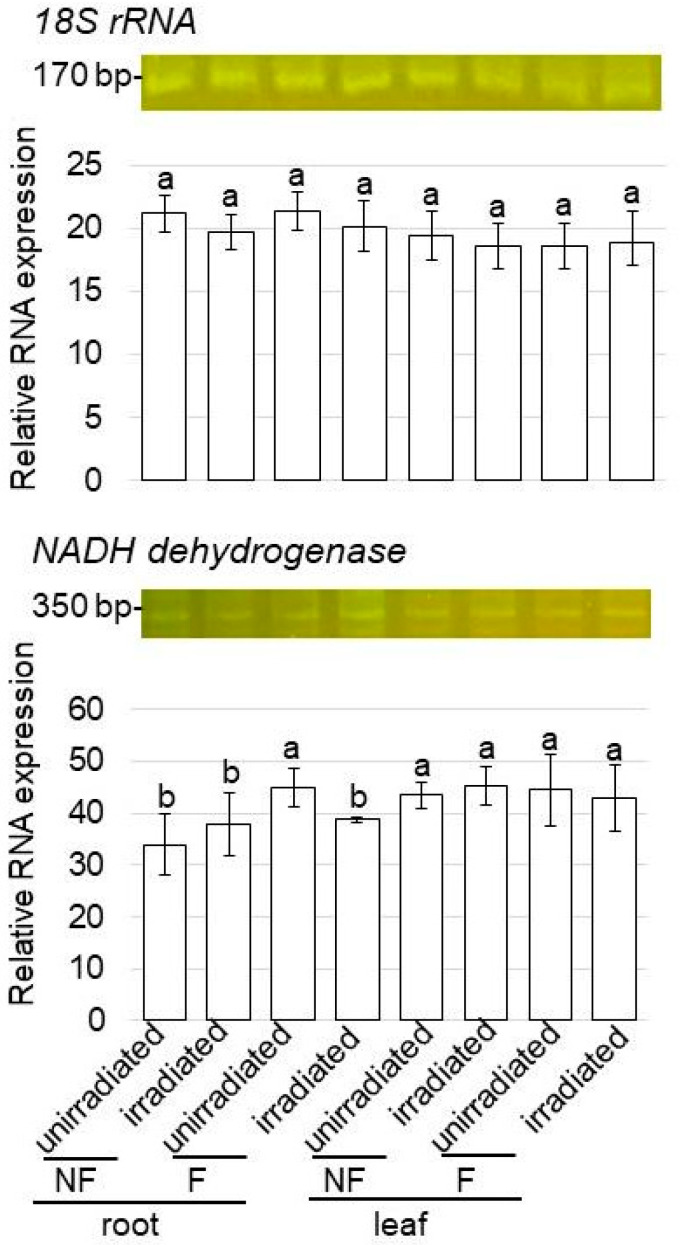
Gene expression of *NADH dehydrogenase* of wheat treated with MMW under flooding stress. RT-PCR analysis of *NADH dehydrogenase* in wheat treated with and without MMW was performed. *NADH dehydrogenase*-specific oligonucleotides were used to amplify transcripts from total RNA isolated from root and leaf wheat. *18S rRNA* was used as an internal control. The data are given as the mean ± SD from three independent biological replicates. Mean values in each point with different letters are significantly different according to one-way ANOVA followed by Tukey’s multiple comparisons (*p* ≤ 0.05).

A PCR analysis of *NADH dehydrogenase* in wheat irradiated with MMW was performed (Figure 6). The expression level of *18S rRNA,* which was used as the internal control, did not change in any treatments (Figure 6). However, *NADH dehydrogenase* was significantly upregulated by flooding stress. Furthermore, following upregulation, the expression recovered to control levels with MMW irradiation, even with flooding stress (Figure 6).

### 2.5. ATP Contents of Wheat Irradiated with MMW under Flooding Stress

Based on the proteomic results, the change in ATP content under flooding stress with MMW irradiation was confirmed with an ATP content assay. Metabolites were extracted from wheat roots treated with or without MMW irradiation under flooding stress. After protein precipitation, the ATP contents were measured (Figure 2).

**Figure 7:**
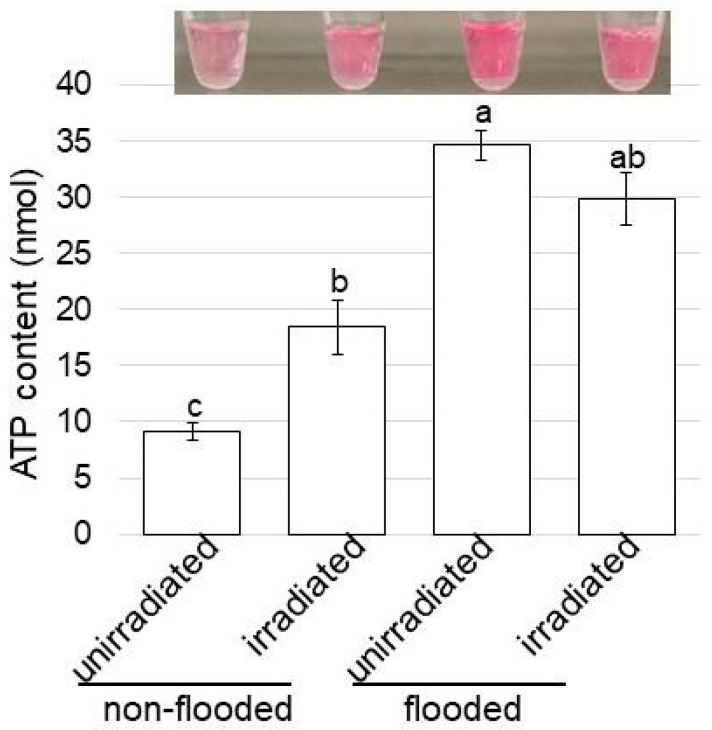
ATP contents of wheat treated with MMW under flooding stress. Wheat seeds were irradiated with and without MMW. Three-day-old wheat was treated with and without flooding stress for 2 days. Metabolites were extracted from the root, and ATP contents were measured for each sample. The data are given as the mean ± SD from three independent biological replicates. Mean values in each point with different letters are significantly different according to one-way ANOVA, followed by Tukey’s multiple comparisons (*p* ≤ 0.05).

When the wheat seeds irradiated with MMW grew, the ATP contents were measured to understand the changes in energy in wheat root under flooding stress (Figure 7). The ATP content significantly increased under flooding stress compared with under the non-flooding condition (Figure 7).

### 2.6. Morphological Effect of Wheat Irradiated with MMW under Flooding Stress

**Figure 8:**
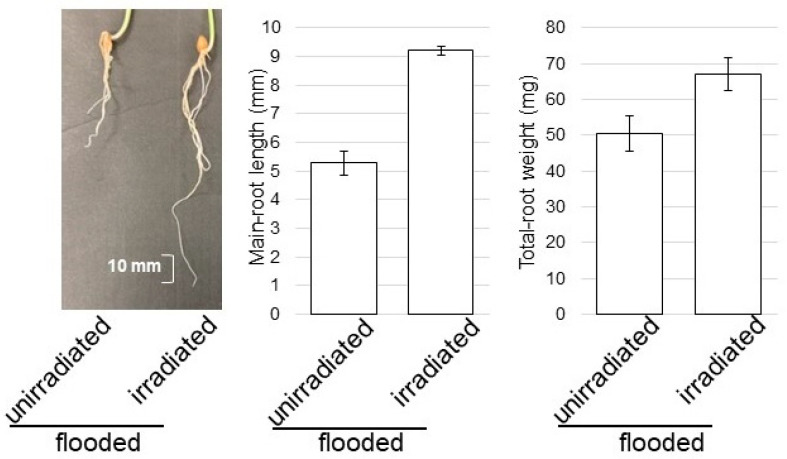
Morphological effect of MMW irradiation under flooding stress. Wheat seeds were irradiated with and without MMW. Three-day-old wheat was treated with flooding stress for 2 days. Main-root length and total-root fresh weight were measured.

To clarify the effect of MMW irradiation on wheat under flooding stress, the change in wheat root treated with MMW was measured with morphological parameters. Under flooding stress, main-root length and total-root fresh weight increased with MMW irradiation (Figure 8). This result indicates that irradiation with MMW was effective in wheat-root growth.

## 3. Discussion

### 3.1. The Role of Plasma Membrane in Wheat, the Seeds of Which Were Irradiated with MMW, under Flooding Stress

MMW introduces thermal energy into the biological systems through incident irradiation, which results in local heating of water molecules at the surface of the cell membranes [19]. MMW irradiation on wheat seeds not only improves germination [12,13] but also enhances the subsequent growth of shoots and grains [14,15]. Furthermore, under flooding stress, MMW affected proteins in wheat grown from irradiated seeds and improved plant growth and flooding tolerance [18]. To understand the mechanism of flooding stress tolerance in MMW-irradiated wheat, membrane proteomics was performed in this study. Seeds with or without MMW irradiation were sown, and the 3-day-old seedlings were treated under flooding stress for 2 days (Figure 2). Membrane fractions were purified from roots, and membrane proteins were extracted and analyzed with LC-MS/MS. After a gene-ontrogy analysis, the proteomic data indicated that the amount of nine proteins decreased and that of three proteins increased in the plasma membrane of wheat with irradiation compared with without irradiation (Figure 3). In plasma membrane proteins, the amount of cellulose synthase decreased under flooding stress; however, following this reduction, the amount recovered to control levels in irradiated wheat, even under flooding stress (Figure 4). A remarkable amount of cellulose [20], which is the main load-bearing component of plant cell walls, is synthesized by cellulose synthase complexes.

The cell wall of plant roots is the first line of defense, as it can strongly protect the plant. The plant cell wall plays a significant role in stress perception by changing and remodeling the plants’ growth strategies in response to stress [21]. The cellulose synthase complex is a sophisticated molecular machinery t hat propels itself forward through the plasma membrane with its own catalytic activity while using cortical microtubules as steering devices [22]. Tetratricopeptide thioredoxin-like proteins function as bridges connecting stress perception with dynamic regulation of cellulose biosynthesis at the plasma membrane [23]. Cell-wall-synthesis-related proteins, such as cinnamyl-alcohol dehydrogenase and cellulose synthase-interactive protein-like proteins, decreased in seedlings grown for 3 days after flooding, indicating that these proteins were associated with the suppression of soybean growth after flooding [24]. Cellulose synthase, auxin, and mitochondrial ribosomal-related genes act as the target genes of miRNAs in the improvement in hypoxic tolerance of cucumber seedlings through exogenous calcium application [25]. These findings, along with the present results, suggest that cellulose synthase may have distinct functions involved in the alteration of cell wall status in seedlings. Furthermore, the decrease in cellulose synthase is directly associated with visible injurious effects, such as the suppression of root growth after flooding. Additionally, MMW irradiation improves wheat growth through cellulose accumulation with an increase in cellulose synthase.

### 3.2. The Role of Vacuolar Membrane in Wheat, the Seeds of Which Were Irradiated with MMW, under Flooding Stress

In the vacuole, two proton pumps exist, which are vacuolar proton translocating pyrophosphatase (V-PPase) and V-ATPase. The former is the dominant proton pump in the early developmental stages of plants such as fruits or organs, whereas the latter takes over in the later stages and becomes the dominant pump [26]. The lack of V-PPase and V-ATPase altered vacuolar morphology and defects in auxin transport [27]. V-ATPase has diverse functions related to plant development/growth, and V-ATPase subunits, such as VAB3, are required for cell growth/ion homeostasis in Arabidopsis [28]. Addressing V-ATPase regulation is a promising approach to modulating its activity to improve stress resistance or to increase yield [29]. In the case of water-deficient conditions, cotton V-ATPase played an important role in conferring resistance to dehydration stress [30]. The loss of VHA-a1, which is V-ATPases in the *trans*-Golgi network, resulted in higher salt sensitivity, cell wall defects, and impaired cell elongation, linking the defects to the *trans*-Golgi network [31]. In this study, in vacuole-membrane proteins, the abundance of V-ATPase increased under flooding stress; however, the amount recovered to control levels in irradiated wheat, even under flooding stress (Figure 4). These findings, along with the present results, suggest that V-ATPase also has diverse functions related to wheat development and growth. Additionally, MMW irradiation might improve wheat growth through the regulation of V-ATPase.

### 3.3. The Role of Endoplasmic Reticulum Membrane in Wheat, the Seeds of Which Were Irradiated with MMW, under Flooding Stress

A 64 kDa protein, which was calnexin, was copurified with the V-ATPase from oat roots [32]. V-ATPase and calnexin indeed interact, although only a small amount of the total V-ATPases is associated with calnexin. In oat, V-ATPase was fully assembled in the endoplasmic reticulum, so the entire biosynthesis of the complex takes place in the endoplasmic reticulum of plant cells where the assembly is assisted by the chaperones calnexin and BiP [33]. In wheat, calnexin also acts as a molecular chaperone of V-ATPase assembly in the endoplasmic reticulum [34]. Because calnexin and BiP are located in the endoplasmic reticulum, V-ATPases might be regulated by canonical quality control and its calnexin/calreticulin cycle [35]. Endoplasmic reticulum mediates protein folding and assembly through a well-coordinated system of chaperones including calnexin, protein disulfide isomerase, and heat shock proteins [36,37]. In this study, although 60 kDa calnexin did not change with any treatments, the amount of 70 kDa calnexin significantly increased with flooding stress compared with in the control (Figure 5). Furthermore, following this accumulation, the amount recovered to control levels in irradiated wheat, even with flooding stress (Figure 5).

In soybean, calnexin accumulation increased in both the wild type and mutant line under flooding stress; however, calreticulin accumulated in only the mutant line under the same condition [38]. The accumulation of calnexin significantly increased in soybean root under flooding stress with or without silver nanoparticles/nicotinic acid/KNO_3_ treatment compared with the case without treatment; however, calreticulin significantly increased under flooding with silver nanoparticles/nicotinic acid/KNO_3_ treatment [39]. Endoplasmic reticulum stress through activation of the misfolded protein response was observed through increases in the levels of calnexin, BiP/GRP78, ERO1-Lα, and protein disulfide isomerase, which may relate to the venetoclax-mediated inhibition of BCL2 in the endoplasmic reticulum [40]. Phosphorylation of the cytoplasmic tail of calnexin controls the endoplasmic reticulum calcium ATPase and, thus, the movement of calcium in and out of the endoplasmic reticulum. Its expression under various stress conditions gives insights into the crosstalk between ER stress and abiotic stress signaling [41]. These findings, along with the present results, suggest that calnexin might increase or be phosphorylated to control misfolding of wheat root proteins under flooding stress. However, calnexin in irradiated wheat might decrease to control levels through the reduction in misfolded proteins.

### 3.4. The Role of Mitochondrial Membrane in Wheat, the Seeds of Which Were Irradiated with MMW, under Flooding Stress

The introduction of endoplasmic reticulum stress from the accumulation of misfolded proteins reveals a much broader functional link between chaperones and mitochondrial metabolism. This cellular condition results in the increased production of reactive oxygen species and activates a cellular signaling mechanism that counteracts the accumulation of misfolded proteins [42]. Calnexin-deficient cells compensated for the loss of this function by partially shifting the energy generation to the glycolytic pathway that showed closer apposition between the endoplasmic reticulum and mitochondria [43]. Calnexin, therefore, controls the cellular energy balance between oxidative phosphorylation and glycolysis. In this study, *NADH dehydrogenase*, which is one of the mitochondrial membrane proteins, was significantly upregulated by flooding stress. Furthermore, following this upregulation, the expression recovered to control levels with MMW irradiation even with flooding stress (Figure 6). Additionally, the ATP content significantly increased under flooding stress compared with under the non-flooding condition (Figure 7).

Cellular energy is provided in the form of ATP and is mainly produced in mitochondria through oxidative phosphorylation. The respiratory chain is composed of four complexes localized in the inner mitochondrial membrane. Complex I and complex II are NADH-ubiquinone oxidoreductase and FADH_2_-ubiquinone oxidoreductase, respectively, both of which transfer electrons from matrix-localized reducing equivalents to ubiquinone [44]. In addition to the classical electron-transport chain consisting of large complexes that transfer electrons from intramitochondrial NADH to oxygen and bind to proton translocation and ATP synthesis, plant mitochondria contain an alternative pathway that does not conserve energy [45,46]. In soybean, aconitase, acyl CoA oxidase, succinate dehydrogenase, and NADH ubiquinone dehydrogenase were upregulated at the transcription level under flooding stress [47]. These results suggest that NADH dehydrogenase might transiently increase to produce ATP in wheat root under flooding stress. Furthermore, the increase in NADH dehydrogenase is not necessary because mitochondria is not damaged in irradiated wheat.

## 4. Materials and Methods

### 4.1. Plant Material, MMW Irradiation, and FloodingTreatment

A Gunn oscillator (J. E. Caristrom, Chicago, IL, USA) was used as the MMW source. The irradiation procedure was described in a previous study [18]. The frequency tuning range of the Gunn oscillator was 79 to 115 GHz, and the output power, which depends on the output frequency, was 7 to 80 mW. The Gunn oscillator was used in free running mode at 110 GHz for the irradiation experiment. The electromagnetic waves emitted from the Gunn oscillator was passed through an isolator. After adjusting the output power using an attenuator, the electromagnetic wave was directed to a free space via a horn antenna with an aperture angle of 17 degrees on each side. By placing a 5 cm diameter Petri dish containing the seeds of wheat (*Triticum aestivum* L. cultivar Nourin 61) at 15 cm from the horn antenna, the MMW irradiation area fully covered the dish. The irradiation time and power were fixed at 20 min and 20 mW, respectively. The average intensity of the electromagnetic waves at the seeds was 0.25 mW/cm^2^. The irradiated electromagnetic wave had a Gaussian distribution, with a maximum intensity of 2.38 times the average intensity at the center of the beam and a minimum intensity of 0.32 times the average intensity at the rim of the beam. The temperature rise in the wheat was estimated to be well below 1 K with the irradiation condition mentioned above.

After irradiation, the seeds were sterilized with a 2% sodium hypochlorite solution, rinsed twice in water, and sown in 400 mL of silica sand in the seedling case. A total of 20 seeds were sown evenly in each seedling case. Wheats were grown at 25 °C and 60% humidity under white fluorescent light (160 µmol/m^2^ s^1^, 16 h light period/day). To induce flooding stress, water was added to 5 cm above the sand surface to immerse 3-day-old wheats in water for 2 days. Irradiated/unirradiated and flooded/non-flooded wheats were collected. For the membrane protein purification and proteomic analysis, roots of 6-day-old wheats were collected. Three independent experiments, in which the seeds were sown on different days, were performed as biological replications for all experiments.

### 4.2. Isolation of Membrane Fractions

All purification procedures were carried out on ice. Membranes were isolated according to the manufacturer’s instructions of the Mem-PER Plus Membrane Protein Extraction Kit (Thermo Fisher Scientific, San Jose, CA, USA) with some modifications. The procedures are described in a previous study [48] (Table S1).

### 4.3. Protein Concentration Measurement

The detergents from the protein extracts were removed using the Pierce Detergent Removal Spin Column (Pierce Biotechnology, Rockford, IL, USA). The method of Bradford [49] was used as the standard to determine the protein concentration with bovine serum albumin.

### 4.4. Protein Preparation for Proteomic Analysis

The quantified proteins (50 µg) were adjusted to a final volume of 100 µL, and the proteins were enriched, reduced, alkylated, and digested. The procedures are described in a previous study [50] (Table S1).

### 4.5. Protein Identification Using Nano-Liquid Chromatography Mass Spectrometry

The liquid chromatography (LC) conditions, as well as the mass spectrometry (MS) acquisition conditions, are described in a previous study [39] (Table S1).

### 4.6. Analysis of MS Data

The MS/MS searches were carried out using SEQUEST HT search algorithms against the UniprotKB *Triticum aestivum* (SwissProt TaxID = 4565) (version 2021-08-20) using Proteome Discoverer 2.5 (version 2.5.0.400; Thermo Fisher Scientific). The procedures are described in a previous study [39] (Table S1).

### 4.7. Differential Analysis of Proteins Using MS Data

Label-free quantification was also performed with Proteome Discoverer 2.2 using precursor ion quantifier nodes. For differential analysis of the relative abundance of peptides and proteins between the samples, the free software PERSEUS (version 1.6.14.0) [51] was used. The procedures are described in a previous study [39] (Table S1).

### 4.8. Immunoblot Analysis

For the immunoblot used as a confirmation experiment, proteins were extracted using a buffer consisting of 50 mM Tris-HCl (pH 6.8), 150 mM NaCl, 1% nonided-P40, 0.5% sodium deoxycholate, 0.1% SDS, and 5% dithiothreitol. An SDS-sample buffer consisting of 60 mM Tris-HCl (pH 6.8), 2% SDS, 10% glycerol, and 5% dithiothreitol was added to the protein extracts [52]. The quantified proteins (10 µg) were separated via electrophoresis on a 10% SDS-polyacrylamide gel and transferred onto a polyvinylidene difluoride (PVDF) membrane using a semidry transfer blotter (Nippon Eido, Tokyo, Japan) [48]. The blotted PVDF membrane was blocked for 5 min in a Bullet Blocking One regent (Nacalai Tesque, Kyoto, Japan). After blocking, the PVDF membrane was cross-reacted with a 1:1000 dilution of the primary antibodies for 1 h at room temperature. As primary antibodies, the anti-H^+^ATPase (Agrisera, Vannas, Sweden), anti-calnexin [53], anti-V-ATPase (Cosmo Bio, Tokyo, Japan), anti-ascorbate peroxidases [54], and cellulose synthetase (Cosmo Bio) antibodies were used. Anti-rabbit IgG conjugated with horseradish peroxidase (BioRad, Hercules, CA, USA) was used as the secondary antibody. After 1 h of incubation, signals were detected using a 3,3′,5,5′-tetramethylbenzidine solution (Nacarai, Kyoto, Japan), following the protocol recommended by the manufacturer. The integrated densities of the bands were calculated using Image J software (version 1.8; National Institutes of Health, Bethesda, MD, USA).

### 4.9. Measurement of ATP Contents

The ATP content was measured using an ATP Colorimetric/Fluorometric Assay Kit (Biovision, Milpitas, CA, USA). A portion (150 mg) of the samples was homogenized in 100 μL of the ADP assay buffer and centrifuged at 16,000× *g* for 10 min at 4 °C. For sample deproteinization and neutralization, the supernatant was treated with a Deproteinizing Sample Preparation Kit (Biovision). Extracts (50 μL) were added to 50 μL of a reaction mixture containing the ADP assay buffer, ADP converter, ADP probe, and ADP developer. After mixing and incubation for 30 min at room temperature in the dark, the absorbance was measured at 570 nm.

### 4.10. RNA Extraction and Polymerase Chain Reaction Analysis

A portion (0.5 g) of the samples was quickly frozen in liquid nitrogen and was ground into a powder with a mortar and pestle. Total RNA was isolated according to a previous procedure [55]. First-strand cDNA was synthesized from 1 µg of total RNA using the iSuperscript Reverse Transcription Supermix (BioRad). Gene-specific primers for 18S rRNA (X02623) (F 5′-TGATTAACAGGGACAGTCGG-3′; R 5′-ACGGTATCTGATCGTCTTCG-3′) and NADH dehydrogenase (A0A3B5YXF8) (F 5′-CTACCTTCCCGTCTCTGTCG-3′; R 5′-GCTCCTGTCACTCGACCTTC-3′) were used to amplify the 200 and 300 bp regions, respectively. Polymerase chain reaction (PCR) cycling was performed as follows using an EmeraldAmp PCR Master Mix (Takara, Tokyo, Japan): 10 s at 98 °C, 30 s at 60 °C, and 30 s at 72 °C (30 cycles). Amplified PCR products were separated by a 2% agarose gel and stained with the Atlas ClearSight Gold DNA stain (BioAtlas, Tartu, Estonia). The integrated densities of the bands were calculated using Image J software.

### 4.11. Statistical Analysis

The statistical significance of data between two groups was analyzed using a Student’s *t*-test. A *p*-value of less than 0.05 was considered statistically significant.

One-way ANOVA followed by Tukey’s multiple comparison was performed among multiple groups using SPSS software (version 22.0, IBM, Armonk, NY, USA). A *p* value < 0.05 was considered statistically significant.

## 5. Conclusions

MMW irradiation of wheat seeds enhanced root growth under flooding stress [18]. To understand its mechanism, in this study, membrane proteomics was performed. Membrane fractions purified from roots were evaluated for their purity. The membrane fraction was rich in H^+^-ATPase and calnexin, which are marker proteins for checking membrane purity. A principal component analysis of the proteomic results indicated that MMW irradiation of seeds affects membrane proteins in grown root. Proteins identified using the proteomic analysis were confirmed using immunoblot and PCR analyses. The key findings were as follows: (i) the abundance of cellulose synthetase, which is a plasma-membrane protein, decreased under flooding stress but increased with MMW irradiation; (ii) the abundance of calnexin and V-ATPase, which are proteins in the endoplasmic reticulum and vacuolar, increased under flooding stress but decreased with MMW irradiation; (iii) *NADH dehydrogenase*, which is located in mitochondria membranes, was upregulated by flooding stress but downregulated by MMW irradiation; and (iv) the ATP content showed a similar trend toward change in *NADH dehydrogenase* expression even under flooding stress. These results suggest that the MMW irradiation of wheat seeds improves root growth by constructing cell walls, reducing the amount of unfolded proteins, regulating waste products, and controlling the ATP content in membranes. Studying this can provide insights into the response of agricultural species to MMW radiation, which in turn can be used to understand their behavior under various environmental and other external conditions.

## Figures and Tables

**Table 1:** List of membrane proteins that significantly changed in wheat root irradiated with MMV compared with that unirradiated under flooding stress.

Accession	Description	Cov	MP	MW [kDa]	calc pI	Ratio	*p*-Value
A0A3B6IP18	Uncharacterized protein	35	14	55.3	7.18	0.010	0.0000
A0A3B6EEJ9	NADH-cytochrome b5 reductase	54	13	33.8	8.51	0.010	0.0000
A0A3B6TGU0	Protein kinase domain-containing protein	10	7	92.4	6.92	0.010	0.0000
A0A3B6HR67	Transmembrane 9 superfamily member	14	6	53.7	6.99	0.010	0.0000
A0A3B6THX1	Phospholipid-transporting ATPase	4	3	122.5	6.02	0.010	0.0000
A0A3B6PIJ7	LEA_2 domain-containing protein	20	3	22.1	8.97	0.010	0.0000
A0A3B6AS01	S-acyltransferase	8	2	35.5	7.62	0.010	0.0000
A0A3B5YXF8	NADH dehydrogenase [ubiquinone] 1 alpha subcomplex assembly factor 3	22	4	19.2	9.01	0.226	0.0000
A0A3B6NKH5	Uncharacterized protein	49	20	50.0	5.69	0.234	0.0000
A0A341Q6B8	Cytochrome b-c1 complex subunit Rieske	26	6	26.5	8.90	0.290	0.0000
A0A3B6I2H7	PHB domain-containing protein	11	3	38.7	8.91	0.296	0.0000
A0A3B6TRE9	L-ascorbate peroxidase	40	11	31.7	7.97	0.310	0.0000
A0A3B5Y6E2	MFS domain-containing protein	3	2	53.9	9.23	0.328	0.0000
A0A3B6AV87	Mitochondrial pyruvate carrier	29	3	11.8	10.01	0.371	0.0008
A0A3B6LTV3	Uncharacterized protein	30	2	10.9	9.25	0.377	0.0001
A0A3B6RDH6	Transmembrane 9 superfamily member	11	6	74.4	6.28	0.389	0.0021
Q06I91	Fasciclin-like protein FLA15	18	4	29.4	8.95	0.446	0.0026
A0A341VXI7	Uncharacterized protein	12	3	30.9	8.15	0.458	0.0031
A0A3B6JIV6	Uncharacterized protein	10	2	28.8	7.31	0.468	0.0213
A0A3B6FTI3	Uncharacterized protein	30	2	7.1	9.29	0.484	0.0007
A0A3B6IMV7	Uncharacterized protein	51	27	80.1	6.64	0.500	0.0037
A0A3B6MTW1	DOMON domain-containing protein	9	2	26.2	9.13	0.513	0.0039
A0A3B6PLV7	AA_permease_C domain-containing protein	4	2	64.0	8.27	0.514	0.0376
A0A1D5YJK2	Signal peptidase complex subunit 3	19	3	18.8	8.18	0.532	0.0029
A0A3B6SMF7	Nicalin	25	13	60.7	6.77	0.552	0.0372
A0A3B6JK94	Conserved oligomeric Golgi complex subunit 6	4	2	76.8	6.25	0.553	0.0350
Q2L9B8	Vacuolar ATP synthase subunit E	44	11	26.1	6.87	0.554	0.0051
A0A3B5YT45	Uncharacterized protein	13	6	74.7	6.32	0.554	0.0242
A0A3B6HW37	UDP-glucose 6-dehydrogenase	39	14	52.8	6.29	0.562	0.0022
A0A3B6MRT6	TPT domain-containing protein	9	2	42.6	10.15	0.572	0.0349
A0A3B5YXX9	Uncharacterized protein	7	4	68.6	6.77	0.580	0.0080
A0A3B6KP84	Cytochrome b-c1 complex subunit 7	47	6	14.5	9.57	0.584	0.0070
A0A3B5Z0L3	PRA1 family protein	12	2	23.1	9.04	0.585	0.0458
A0A3B6MVJ2	Transmembrane 9 superfamily member	19	11	73.3	7.21	0.591	0.0144
A0A3B6IWB7	Phytocyanin domain-containing protein	18	4	21.1	6.51	0.606	0.0161
A0A2X0S7E3	Prohibitin	38	7	30.6	6.98	0.611	0.0208
A0A3B6AY90	Phytocyanin domain-containing protein	17	3	20.5	8.81	0.614	0.0269
A0A341W842	Plug translocon domain-containing protein	12	6	52.5	8.98	0.624	0.0229
A0A1D5V328	Genome assembly, chromosome: II	38	5	18.1	4.41	0.626	0.0300
A0A1D5XTG1	ER membrane protein complex subunit 4	23	3	19.2	9.04	0.631	0.0398
A0A3B6U6Q6	Dolichyl-diphosphooligosaccharide-protein glycosyltransferase subunit 1	19	6	52.5	7.69	0.635	0.0421
A0A3B6IUL0	Protein kinase domain-containing protein	3	3	91.1	7.17	1.467	0.0159
A0A3B6QDR2	Uncharacterized protein	44	22	60.1	4.81	1.552	0.0463
A0A3B6IRG1	Glucan endo-1,3-beta-D-glucosidase	5	2	48.9	6.32	1.592	0.0290
A0A3B6TMJ9	Protein kinase domain-containing protein	2	2	111.5	5.90	1.785	0.0047
A0A3B5YYP9	Uncharacterized protein	30	2	13.2	8.94	1.916	0.0014
A0A3B5ZXA9	CASP-like protein	11	2	19.8	9.51	2.047	0.0312
A0A3B6JL36	SHSP domain-containing protein	11	2	24.1	5.40	2.456	0.0001
A0A3B6LI60	1-acylglycerol-3-phosphate O-acyltransferase	8	2	38.8	9.88	3.664	0.0000
A0A3B6MMS9	1-acylglycerol-3-phosphate O-acyltransferase	9	2	38.8	9.74	5.547	0.0000
A0A3B6AX46	Succinate dehydrogenase [ubiquinone] flavoprotein subunit, mitochondrial	31	13	68.0	6.68	10.736	0.0000

Cov, coverage (%); MP, matched peptides; MW, molecular weight; calc pI, calculated pI; Ratio, treatment/control.

## Data Availability

The MS data, RAW data, peak lists, and result files have been deposited in the ProteomeXchange Consortium [56] via the jPOST [57] partner repository under the dataset identifier PXD040924.

## Supplementary Materials

The supporting information can be downloaded at https://www.mdpi.com/article/10.3390/ijms24109014/s1.
