# Supplementary material for: Membrane Proteomics to Understand Enhancement Effects of Millimeter-Wave Irradiation on Wheat Root under Flooding Stress
Source: Int J Mol Sci. 2023 May 19;24(10):9014. doi: 10.3390/ijms24109014 (PMC10219106; doi:10.3390/ijms24109014)
Supplement: Supplementary file 1 [file ijms-24-09014-s001.zip › Supplemental Figures.pdf]

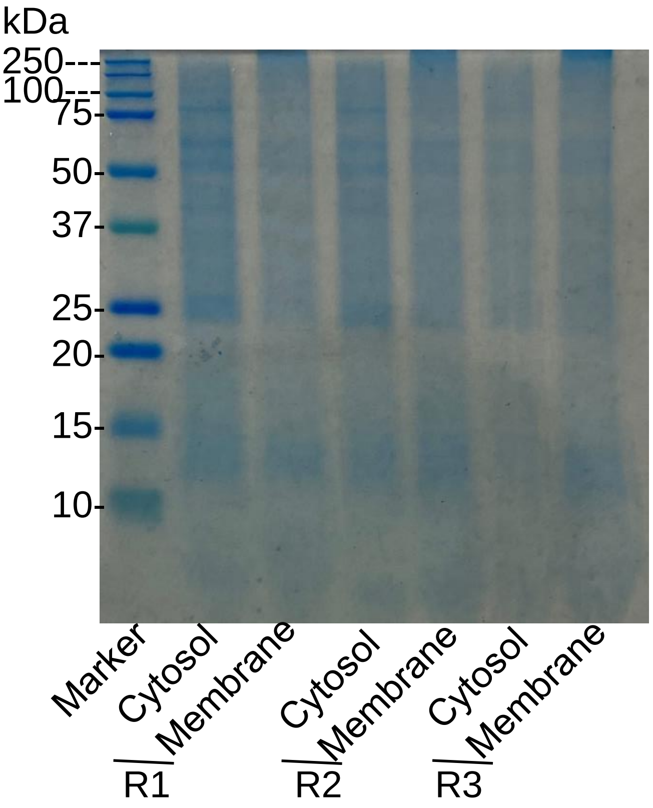

Figure S1. The Coomassie brilliant blue staining patterns of proteins used for immuno-blot analysis used in purity check. Experiments were performed with biologically triplicates for each fraction (R1, R2, and R3). Quantified proteins (10 µg) of membrane fraction purified from root were separated by electrophoresis on a 10% SDS-polyacrylamide. Coomassie brilliant blue staining was used as a loading control.

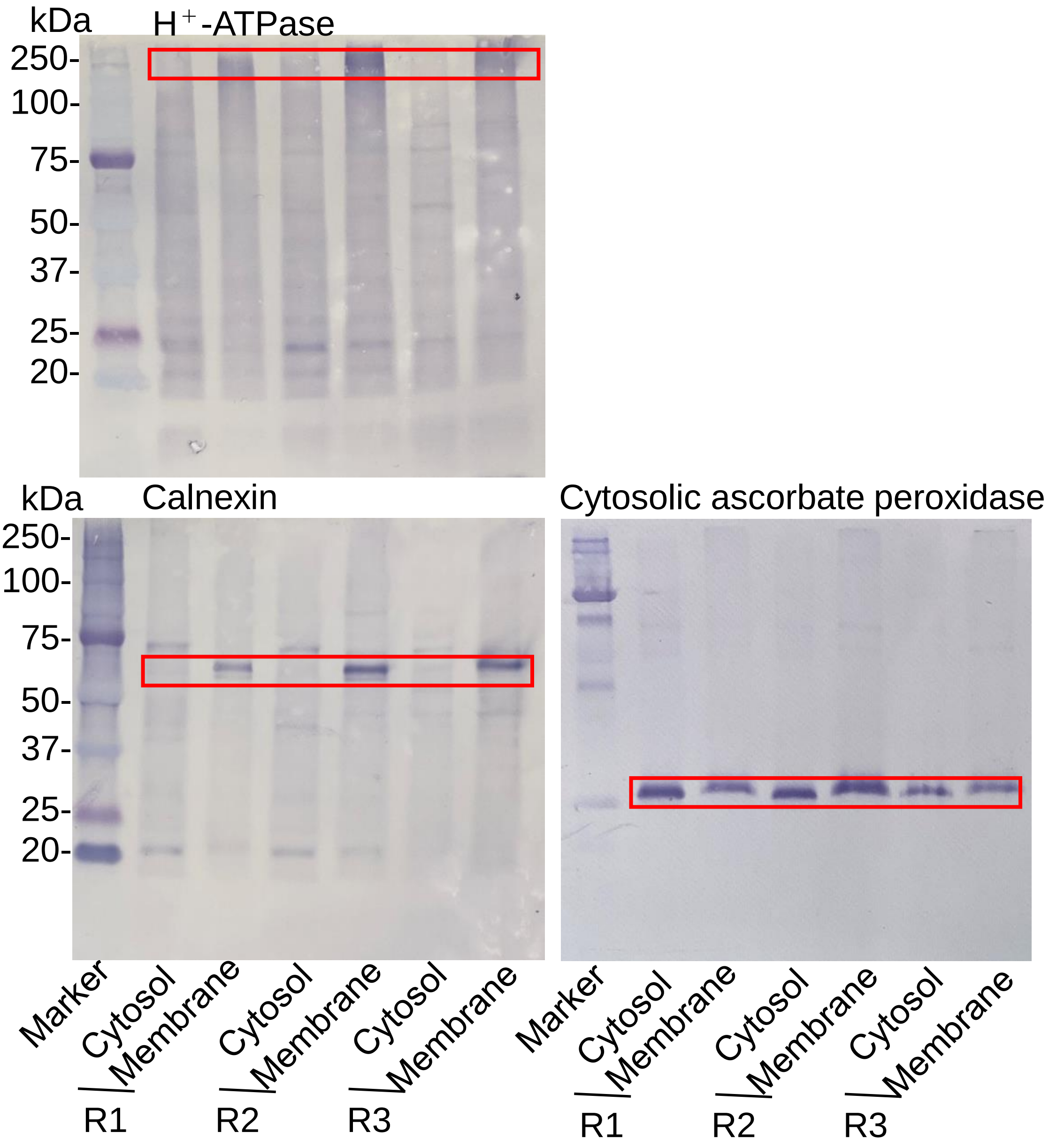

Figure S2. Blots of the entire membrane with antibodies used in purity check, which are used in Figure 2. Experiments were performed with biologically triplicates for each fraction (R1, R2, and R3). Quantified proteins (10 μg) of membrane fraction purified from root were separated by electrophoresis on a 10% SDS-polyacrylamide. The PVDF membrane was cross-reacted with anti-H^{+}-ATPase, calnexin, and cytosolic ascorbate peroxidase antibody.

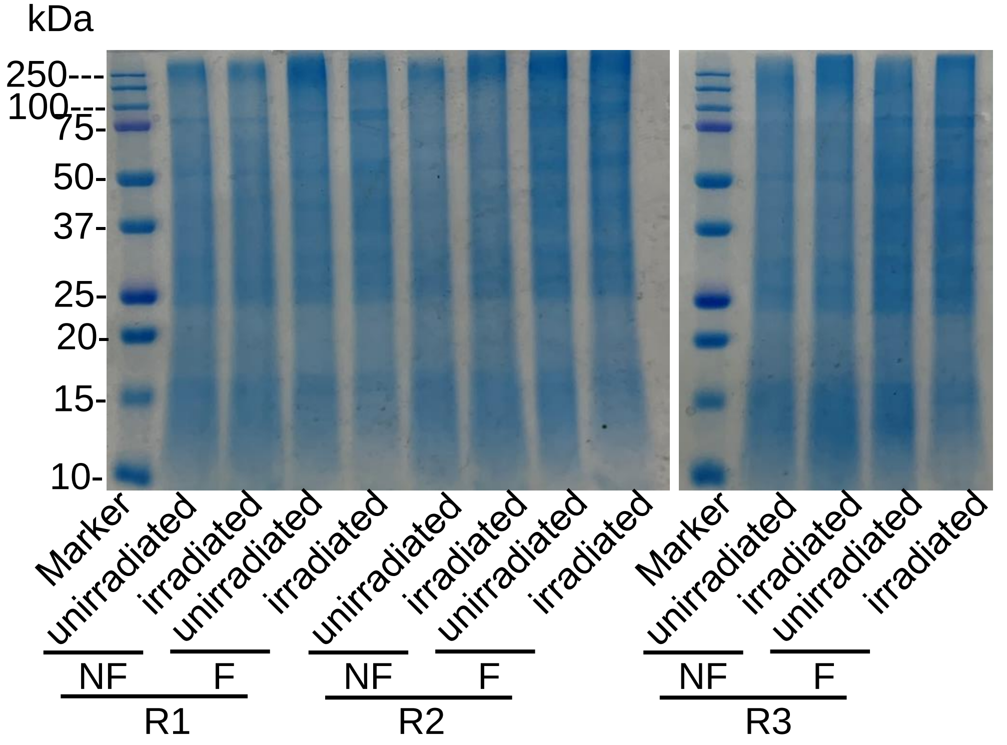

Figure S3. The Coomassie brilliant blue staining patterns of proteins used for immuno-blot analysis used in confirmation experiments. Experiments were performed with biologically triplicates (R1, R2, and R3). Quantified proteins (10 µg) from root were separated by electrophoresis on a 10% SDS-polyacrylamide. Coomassie brilliant blue staining was used as a loading control. NF and F mean non-flooding and flooding, respectively.

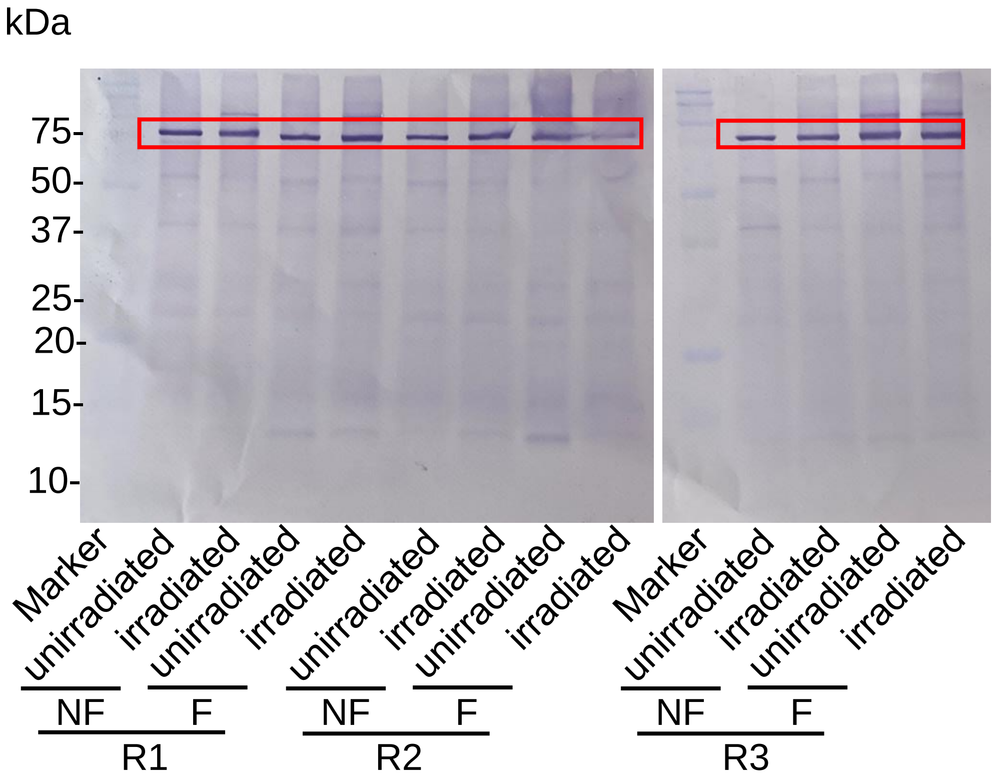

Figure S4. Blots of the entire membrane with anti-cellulose synthetase antibody, which are used in Figure 4A. Proteins (10 μg) extracted from root were separated on SDS-polyacrylamide gel and transferred on to PVDF membrane. PVDF membrane was cross-reacted with anti-cellurose synthetase antibody. Data show 3 biological replications (R1, R2, and R3). NF and F mean non-flooding and flooding, respectively.

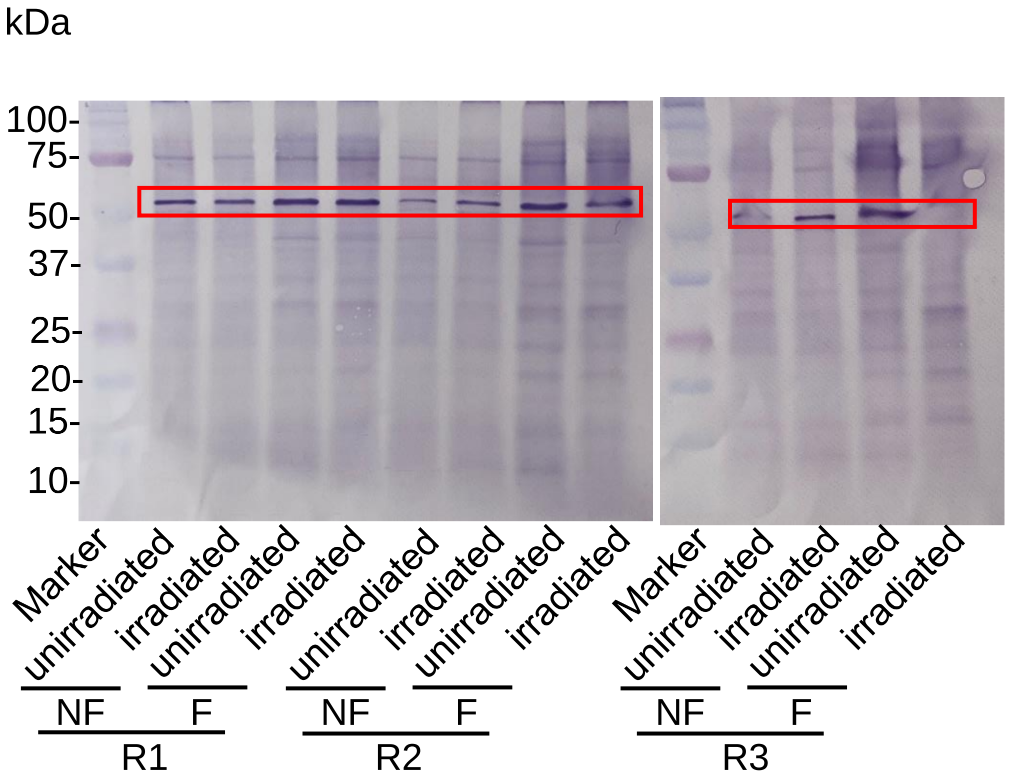

Figure S5. Blots of the entire membrane with anti-V-ATPase antibody, which are used in Figure 4B. Proteins (10 μg) extracted from root were separated on SDS-polyacrylamide gel and transferred on to polyvinylidene difluoride membrane. The polyvinylidene difluoride membrane was cross-reacted with anti-V-ATPase antibody. Data show 3 biological replicates (R1, R2, and R3). NF and F mean non-flooding and flooding, respectively.

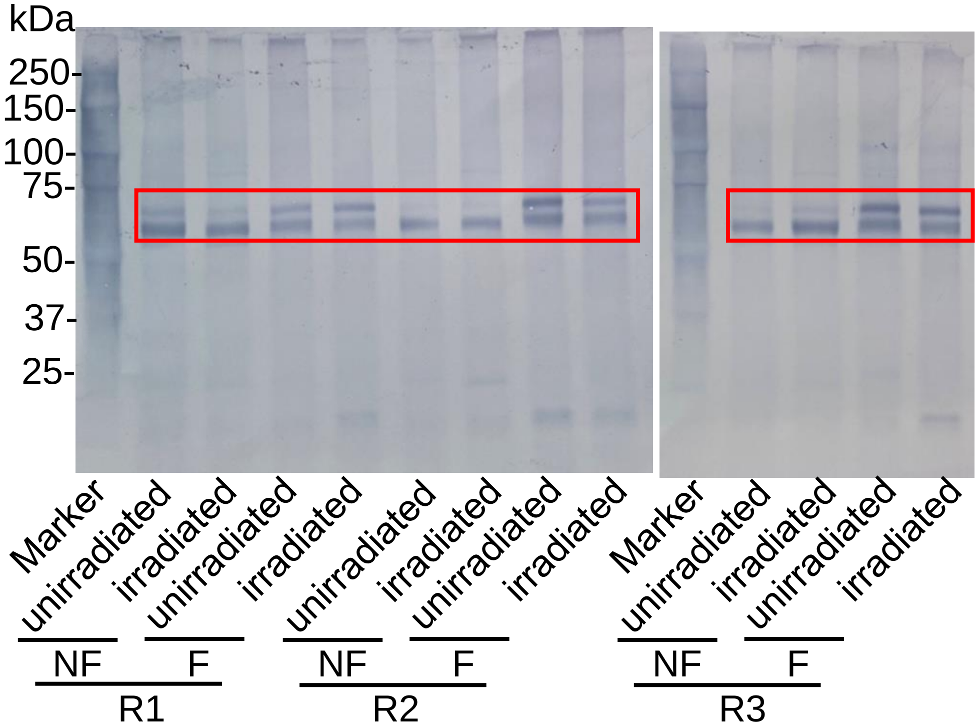

Figure S6. Blots of the entire membrane with anti-calnexin antibody, which are used in Figure 5. Proteins (10 μg) extracted from root were separated on SDS-polyacrylamide gel and transferred on to polyvinylidene difluoride membrane. The polyvinylidene difluoride membrane was cross-reacted with anti-calnexin antibody. Data show 3 biological replicates (R1, R2, and R3). NF and F mean non-flooding and flooding, respectively.
