# Supplementary material for: Membrane Proteomics to Understand Enhancement Effects of Millimeter-Wave Irradiation on Wheat Root under Flooding Stress
Source: Int J Mol Sci. 2023 May 19;24(10):9014. doi: 10.3390/ijms24109014 (PMC10219106; doi:10.3390/ijms24109014)
Supplement: Supplementary file 1 [file ijms-24-09014-s001.zip › Supplemental Tables.pdf]

Table S1. Materials and methods.

Isolation of membrane fractions	All purification procedures were carried out on ice. Membranes were isolated according to the manufacturer's instructions of Mem-PER Plus Membrane Protein Extraction Kit (Thermo Fisher Scientific, San Jose, CA, USA) with some modifications. The procedures are described in the previous study (Murashita et al., 2021). Briefly, a portion (2.0 g) of samples was chopped and ground in 1 mL of permeabilization buffer using a mortar and pestle. Homogenates were transferred to a tube, and incubated for 10 min at 4°C with constant mixing and centrifuged at 16,000 x g for 15 min at 4°C to pellet permeabilized cells. The supernatant containing cytosolic proteins was removed and transferred to a new tube. The pellet was resuspended in 0.5 mL of solubilization buffer. After pipetting, a homogeneous suspension was obtained and incubated for 30 min at 4°C with constant mixing. After centrifugation at 16,000 x g for 15 min at 4°C, supernatant containing solubilized membrane and membrane-associated proteins were sonicated with lysis buffer consisting of 7 M urea, 2 M thiourea, 5% CHAPS, and 2 mM tributylphosphine. After sonication, the homogenate was centrifuged at 12,000 x g for 30 min at 4°C, and the supernatant was collected as membrane proteins.
Protein enrichment, reduction, alkylation, and digestion	Quantified proteins (50 µg) were adjusted to a final volume of 100 µL; and proteins were enriched, reduced alkylated, and digested. The procedures are described in the previous study (Komatsu et al., 2013). Briefly, Methanol (400 µL) was added to each sample and mixed before addition of 100 µL of chloroform and 300 µL of water. After mixing and centrifugation at 20,000 x g for 10 min to achieve phase separation, the upper phase was discarded and 300 µL of methanol was added to the lower phase, and then centrifuged at 20,000 x g for 10 min. The pellet was collected and resuspended in 50 mM ammonium bicarbonate. It was reduced with 50 mM dithiothreitol for 30 min at 56°C and alkylated with 50 mM iodoacetamide for 30 min at 37°C. Alkylated proteins were digested with trypsin and lysyl endopeptidase (Wako, Osaka, Japan) at a 1:100 enzyme/protein ratio for 16 h at 37°C. Peptides were desalted with MonoSpin C18 Column (GL Sciences, Tokyo, Japan) and acidified with 1% trifluoroacetic acid.
Protein identification using nano-liquid chromatography mass spectrometry	The liquid chromatography (LC) conditions as well as the mass spectrometry (MS) acquisition conditions were described in the previous study (Hashimoto et al., 2020). Briefly, the peptides were loaded onto the LC system (EASY-nLC 1200; Thermo Fisher Scientific) equilibrated with 0.1% formic acid and eluted with a linear acetonitrile gradient (0-35%) in 0.1% formic acid at a flow rate of 300 nL min^{-1}. The eluted peptides were loaded and separated on the Aurora column (25 cm x 75 µm ID, 1.6 mm C18; Ionoptics) with a spray voltage of 1.5 kV (Ion Transfer Tube temperature: 275°C). The peptide ions were detected using MS (Orbitrap Fusion ETD MS; Thermo Fisher Scientific) in the data-dependent acquisition mode with the installed Xcalibur software (version 4.0; Thermo Fisher Scientific). Full-scan mass spectra were acquired in the MS over 375-1,500 m/z with resolution of 120,000. The most intense precursor ions were selected for collision-induced fragmentation in the linear ion trap at normalized collision energy of 35%. Dynamic exclusion was employed within 60 sec to prevent repetitive selection of peptides.
Mass spectrometry data analysis	The MS/MS searches were carried out using SEQUEST HT search algorithms against the UniprotKB *Triticum aestivum* (SwissProt TaxID = 4565) (version 2021-08-20) using Proteome Discoverer 2.5 (version 2.5.0.400; Thermo Fisher Scientific). The procedures are described in the previous study (Hashimoto et al., 2020). Briefly, The workflow for both algorithms included spectrum files RC, spectrum selector, SEQUEST HT search nodes, percolator, ptmRS, and minor feature detector nodes. Oxidation of methionine was set as a variable modification and carbamidomethylation of cysteine was set as a fixed modification. Mass tolerances in MS and MS/MS were set at 10 ppm and 0.6 Da, respectively. Trypsin was specified as protease and a maximum of 2 missed cleavage was allowed. Target-decoy database searches used for calculation of false discovery rate, which was set at 1% for peptide identification.
Differential analysis of proteins using mass spectrometry data	Label-free quantification was also performed with Proteome Discoverer 2.2 using precursor ions quantifiler nodes. For differential analysis of the relative abundance of peptides and proteins between samples, the free software PERSEUS (version 1.6.14.0) (Tyanova et al., 2016) was used. The procedures are described in the previous study (Hashimoto et al., 2020). Briefly, abundances of proteins and peptides abundances were transferred into log2 scale. Three biological replicates of each sample were grouped and a minimum of 3 valid values were required in at least one group. Normalization of the abundances was performed to subtract the median of each sample. Missing values were imputed based on a normal distribution (width = 0.3, down-shift = 1.8). Significance was assessed using Student's *t*-test analysis. Principal-component analysis was performed with Proteome Discoverer 2.5. The sequences of the differentially accumulated proteins were subjected to a BLAST query against the gene ontology database (http://www.geneontology.org/).

References

Murashita, Y.; Nishiuchi, T.; Rehman, S.U, Komatsu, S. Subcellular proteomics to understand promotive effect of plant-derived smoke solution on soybean root. *Proteomes* **2021,** *9,* 39.

Komatsu, S.; Han, C.; Nanjo, Y.; Altaf-Un-Nahar, M.; Wang, K.; He, D.; Yang, P. Label-free quantitative proteomic analysis of abscisic acid effect in early-stage soybean under flooding. *J Proteome Res* **2013**, *12,* 4769-4784.

Hashimoto, T.; Mustafa, G.; Nishiuchi, T.; Komatsu, S. Comparative analysis of the effect of inorganic and organic chemicals with silver nanoparticles on soybean under flooding stress. *Int J Mol Sci* **2020**, *21*, 1300.

Tyanova, S.; Temu, T.; Cox, J. The MaxQuant computational platform for mass spectrometry-based shotgun proteomics. *Nat Protoc* **2016,** *11,* 2301-2319.

Table S2. List of changed membrane proteins in wheat-root irradiated with millimeter waves compared with unirradiated under flooding stress.

Accession	Description	MP	MW [kDa]	Calc. pI	Abundance Ratio	*P*-Value
A0A3B6JJ96	UDP-arabinopyranose mutase	21	41.4	6.19	0.01	0.0000
A0A3B5YTL5	Asparagine--tRNA ligase	19	63.2	6.09	0.01	0.0000
A0A3B5XX47	Uncharacterized protein	17	73.2	5.19	0.01	0.0000
A0A3B6QEK4	3-hydroxyacyl-CoA dehydrogenase	15	78.7	9.16	0.01	0.0000
A0A3B6QI71	Adenosine kinase	15	37.2	5.1	0.01	0.0000
A0A3B6IP18	Uncharacterized protein	14	55.3	7.18	0.01	0.0000
A0A3B6QKL4	Epimerase domain-containing protein	14	44.6	9.35	0.01	0.0000
A0A3B6EEJ9	NADH-cytochrome b5 reductase	13	33.8	8.51	0.01	0.0000
A0A3B6ITS7	Uncharacterized protein	9	49.9	6.38	0.01	0.0000
A0A3B6U1P5	Uncharacterized protein	9	40.6	7.08	0.01	0.0000
A0A3B6KG18	Beta-galactosidase	8	99.4	6.05	0.01	0.0000
A0A3B6QKT9	Oxidored_FMN domain-containing protein	8	40.6	6.62	0.01	0.0000
A0A3B6TGU0	Protein kinase domain-containing protein	7	92.4	6.92	0.01	0.0000
A0A1D5UM13	Genome assembly, chromosome: II	6	87.5	4.98	0.01	0.0000
A0A3B6NIJ2	Uncharacterized protein	6	40.3	5.74	0.01	0.0000
A0A3B6N1S1	ACB domain-containing protein	6	72.6	5.15	0.01	0.0000
A0A3B6MYB5	Peroxidase	6	34.2	6.4	0.01	0.0000
A0A3B6HR67	Transmembrane 9 superfamily member	6	53.7	6.99	0.01	0.0000
A0A3B6SP41	S5 DRBM domain-containing protein	5	59	6.39	0.01	0.0000
A0A3B6PQW3	Thioredoxin reductase	5	34.9	6.23	0.01	0.0000
A0A3B6HX62	Tetratricopeptide repeat protein 38	4	52.9	5.57	0.01	0.0000
A0A077RXT9	LigB domain-containing protein	4	33.6	7.44	0.01	0.0000
A0A3B6JKY4	Uncharacterized protein	4	26.5	7.65	0.01	0.0000
A0A3B6H355	Peptidase A1 domain-containing protein	3	43.7	9.2	0.01	0.0000
A0A3B6THX1	Phospholipid-transporting ATPase	3	122.5	6.02	0.01	0.0000
A0A3B6JMJ4	Uncharacterized protein	3	27.8	4.96	0.01	0.0000
A0A3B6PIJ7	LEA_2 domain-containing protein	3	22.1	8.97	0.01	0.0000
A0A3B6TF02	YTH domain-containing protein	2	69	5.83	0.01	0.0000
A0A3B6LZ26	Protein kinase domain-containing protein	2	133.7	5.48	0.01	0.0000
A0A3B6JHQ2	Peptidase A1 domain-containing protein	2	46	5.16	0.01	0.0000
A0A3B6EQB1	PUM-HD domain-containing protein	2	110.8	5.9	0.01	0.0000
A0A3B6RNS3	Uncharacterized protein	2	49.8	6.01	0.01	0.0000
A0A3B6AS01	S-acyltransferase	2	35.5	7.62	0.01	0.0000
A0A3B6AQ93	Glyco_hydro_18 domain-containing protein	2	33.3	9.1	0.01	0.0000
A0A3B6KR72	H15 domain-containing protein	2	29.3	10.76	0.01	0.0000
A0A3B6KCP1	AB hydrolase-1 domain-containing protein	2	37.1	5.53	0.01	0.0000
A0A3B6JNK9	GRAM domain-containing protein	2	27.9	7.97	0.01	0.0000
A0A3B6RG09	Uncharacterized protein	2	41	5.02	0.01	0.0000
A0A3B6GQT5	Uncharacterized protein	2	66.4	10.24	0.01	0.0000
A0A3B5Y0B1	Leucyl-tRNA synthetase	2	123.5	7.14	0.01	0.0000
A0A3B6CF83	FAD-binding PCMH-type domain-containing protein	5	66.7	6.86	0.038	0.0000
A0A3B6JKZ7	S1 motif domain-containing protein	7	38.2	5.48	0.064	0.0000
A0A3B5Z0H5	Uncharacterized protein	2	42.4	6.46	0.078	0.0000

A0A3B6IQB1	NAC-A/B domain-containing protein	2	22.4	4.4	0.083	0.0000
A0A3B6KJX5	HATPase_c domain-containing protein	16	88.3	4.98	0.154	0.0000
A0A3B6KT52	RNB domain-containing protein	2	74.3	6.58	0.191	0.0000
W5DEJ5	Ribosomal_L28e domain-containing protein	3	16.2	10.32	0.212	0.0000
A0A3B6N0B4	CMP/dCMP-type deaminase domain-containing protein	2	23.2	6.62	0.216	0.0000
A0A3B5YXF8	NADH dehydrogenase [ubiquinone]	4	19.2	9.01	0.226	0.0000
A0A3B6NKH5	Uncharacterized protein	20	50	5.69	0.234	0.0000
A0A3B6JIQ3	VOC domain-containing protein	4	15.3	6.32	0.246	0.0000
A0A3B5Z5T0	Malate dehydrogenase	12	35.5	8.38	0.253	0.0000
A0A1D6DK86	40S ribosomal protein S24	4	15.7	10.71	0.262	0.0000
A0A3B6KSS8	PfkB domain-containing protein	9	35.7	5.22	0.264	0.0000
A0A3B6I2T7	Epimerase domain-containing protein	3	23.6	8.06	0.264	0.0000
A0A3B6KQL2	SERPIN domain-containing protein	2	43.1	5.87	0.278	0.0000
A0A341Q6B8	Cytochrome b-c1 complex subunit Rieske, mitochondrial	6	26.5	8.9	0.29	0.0000
A0A3B6I2H7	PHB domain-containing protein	3	38.7	8.91	0.296	0.0000
A0A3B6RRE3	Histone H2A	2	17.4	10.56	0.299	0.0000
A0A3B6GP60	NAD(P)H dehydrogenase (quinone)	3	22.5	6.54	0.308	0.0002
A0A3B6TRE9	L-ascorbate peroxidase	11	31.7	7.97	0.31	0.0000
A0A3B6MP76	Uncharacterized protein	9	20	8.02	0.311	0.0000
A0A3B6JRN2	Protein kinase domain-containing protein	3	33.9	6.96	0.322	0.0000
A0A3B6RID6	Uncharacterized protein	14	44	8.05	0.323	0.0000
A0A3B5Y6E2	MFS domain-containing protein	2	53.9	9.23	0.328	0.0000
A0A3B6U9U8	Small nuclear ribonucleoprotein E	2	10.2	9.52	0.33	0.0000
A0A3B6ISB7	UDP-arabinopyranose mutase	21	41.5	6.19	0.335	0.0048
A0A3B6KJC5	Phenylalanine--tRNA ligase	6	56.3	6.38	0.336	0.0001
A0A3B5Z6K1	FAD-binding PCMH-type domain-containing protein	3	59.5	8.25	0.336	0.0009
A0A3B6IV48	Aconitate hydratase	25	86.6	6.4	0.36	0.0391
A0A3B6NJ31	Aspartate aminotransferase	17	47.3	6.99	0.365	0.0000
A0A3B6KNY0	HMG box domain-containing protein	2	13.8	8.92	0.37	0.0024
A0A3B6AV87	Mitochondrial pyruvate carrier	3	11.8	10.01	0.371	0.0008
A0A3B6LTV3	Uncharacterized protein	2	10.9	9.25	0.377	0.0001
A0A1D5YNP5	Small nuclear ribonucleoprotein Sm D3	2	14.5	11.06	0.379	0.0002
A0A3B6RDH6	Transmembrane 9 superfamily member	6	74.4	6.28	0.389	0.0021
A0A3B5XXB2	Glutathione transferase	6	25	6.16	0.394	0.0014
W5GEL1	Proteasome subunit beta	3	22.8	5.44	0.396	0.0000
A0A3B6ILF8	IGR domain-containing protein	3	11.9	10.33	0.404	0.0000
A0A3B6QIS0	Uncharacterized protein	3	33.2	9.09	0.404	0.0022
A0A3B6A2W3	C2H2-type domain-containing protein	5	33.6	4.81	0.412	0.0000
A0A3B6JDJ4	Pyruvate kinase	2	58.9	6.06	0.413	0.0000
A0A3B6IU05	NAD(P)H dehydrogenase (quinone)	6	21.4	7.21	0.414	0.0405
A0A3B6JGN4	Uncharacterized protein	2	31.3	8.06	0.416	0.0269
A0A3B5XZC6	Nascent polypeptide-associated complex subunit beta	5	18.2	7.25	0.421	0.0000
A0A077RVE4	Uncharacterized protein	2	28.9	4.84	0.422	0.0003
A0A3B6QAJ9	Uncharacterized protein	18	27.1	8.12	0.426	0.0000
A0A3B6GWS9	Malate dehydrogenase	10	35.4	8.37	0.426	0.0000
A0A3B6AWA4	Phytocyanin domain-containing protein	2	19.1	9.32	0.429	0.0008

A0A3B6SFH1	Peroxidase	4	37.5	6.77	0.435	0.0007
A0A3B6IZ21	PCI domain-containing protein	16	49.8	6.57	0.437	0.0001
W5ADS2	Ubiquitin	6	17.7	9.8	0.438	0.0002
A0A3B6TJC7	Uncharacterized protein	6	33.8	5.95	0.443	0.0167
U5MY58	Monodehydroasorbate reductase	9	46.7	5.19	0.444	0.0000
Q06I91	Fasciclin-like protein FLA15	4	29.4	8.95	0.446	0.0026
A0A3B6EGC8	Glutaredoxin domain-containing protein	3	20.4	7.28	0.449	0.0481
A0A3B6GU52	Glycine cleavage system P protein	23	111	6.9	0.455	0.0033
A0A3B6NJ70	Acetyltransferase component of pyruvate dehydrogenase complex	16	57.1	8.15	0.455	0.0013
A0A3B6PH36	Uncharacterized protein	11	52.5	7.06	0.455	0.0153
A0A3B6KHQ0	Fe2OG dioxygenase domain-containing protein	2	32.4	5.27	0.455	0.0009
A0A341VXI7	Uncharacterized protein	3	30.9	8.15	0.458	0.0031
W5C0N6	Germin-like protein	2	27.2	8.87	0.458	0.0178
A0A077RQG5	Uncharacterized protein	3	58.7	6.74	0.46	0.0407
A0A3B6LU46	Uncharacterized protein	2	6.4	9.91	0.461	0.0141
A0A3B6HUP2	CCT-theta	19	57.9	5.48	0.464	0.0443
A0A3B6JIR3	Uncharacterized protein	25	71.3	5.21	0.465	0.0009
A0A3B6NTA2	Uncharacterized protein	4	36.3	9.22	0.468	0.0186
A0A3B6JIV6	Uncharacterized protein	2	28.8	7.31	0.468	0.0213
A0A3B6SKM7	Uncharacterized protein	12	16.7	5.16	0.469	0.0186
A0A3B6B4E1	Germin-like protein	2	27.2	8.85	0.473	0.0055
W5HFJ0	Uncharacterized protein	3	13.8	9.94	0.476	0.0000
A0A3B6C7W0	Protein-serine/threonine phosphatase	3	30.6	6	0.476	0.0056
A0A1D6RLV5	Iso_dh domain-containing protein	6	39.4	6.73	0.481	0.0062
A0A3B6FTI3	Uncharacterized protein	2	7.1	9.29	0.484	0.0007
A0A3B6SBW2	Uncharacterized protein	11	60.8	5.05	0.487	0.0016
A0A3B5ZMD5	Oxidored_FMN domain-containing protein	13	40.3	6.34	0.49	0.0019
A0A3B6B9F6	DUF3700 domain-containing protein	10	28.1	7.39	0.49	0.0001
W5D7B8	60S ribosomal protein L18a	11	21.3	10.4	0.496	0.0001
A0A3B6IMV7	Uncharacterized protein	27	80.1	6.64	0.5	0.0037
A0A3B5Y7Z1	Malate dehydrogenase	12	35.5	8.38	0.5	0.0001
A0A3B6NRK4	Uncharacterized protein	13	137.7	5.73	0.507	0.0481
A0A3B6KFB8	NAD(P)-bd_dom domain-containing protein	5	31.2	9.57	0.511	0.0004
A0A3B6B298	Thioredoxin domain-containing protein	2	19.5	9	0.512	0.0037
A0A3B6MTW1	DOMON domain-containing protein	2	26.2	9.13	0.513	0.0039
A0A3B6PLV7	AA_permease_C domain-containing protein	2	64	8.27	0.514	0.0376
A0A3B6KAJ3	Phosphopyruvate hydratase	24	48	5.68	0.515	0.0009
A0A3B6NV46	Uncharacterized protein	11	47.8	8.1	0.516	0.0002
A0A3B6HYW3	Nascent polypeptide-associated complex subunit beta	6	17.3	8.85	0.524	0.0006
A0A3B6MY06	Uncharacterized protein	9	17.7	10.74	0.526	0.0008
A0A341ZKV6	Pept_C1 domain-containing protein	5	20.9	4.79	0.527	0.0004
W5G990	Uncharacterized protein	5	14.1	9.89	0.527	0.0006
Q3S4I1	Eukaryotic translation initiation factor 5A	6	17.4	6.14	0.53	0.0005
A0A3B6PLJ7	Glycine cleavage system H protein	2	17	5.08	0.53	0.0056
A0A1D5YJK2	Signal peptidase complex subunit 3	3	18.8	8.18	0.532	0.0029

A0A3B6EN11	Methanethiol oxidase	3	54.1	5.81	0.538	0.0375
W5BSX7	Genome assembly, chromosome: II	2	20.4	9.79	0.54	0.0012
W5A9E1	Ubiquitin	6	17.6	9.8	0.544	0.0014
A0A3B6H0W1	Transaldolase	17	44.4	5.54	0.546	0.0022
A0A3B6PDY8	Histone H2B	4	15.6	10.21	0.548	0.0013
A0A3B6SMF7	Nicalin	13	60.7	6.77	0.552	0.0372
A0A3B6ITU8	CCT-beta	14	54.4	6.13	0.553	0.0206
A0A3B6JCX3	Uncharacterized protein	6	48.3	5.39	0.553	0.0017
A0A3B5ZWB5	Nucleoside diphosphate kinase	6	16.7	6.8	0.553	0.0206
A0A3B6JK94	Conserved oligomeric Golgi complex subunit 6	2	76.8	6.25	0.553	0.0350
Q2L9B8	Vacuolar ATP synthase subunit E	11	26.1	6.87	0.554	0.0051
A0A3B5YT45	Uncharacterized protein	6	74.7	6.32	0.554	0.0242
A0A3B6EL93	Ribosomal_L28e domain-containing protein	3	16.2	10.32	0.554	0.0052
A0A3B6LIR6	Uncharacterized protein	13	73.5	5.15	0.555	0.0027
A0A3B6HW37	UDP-glucose 6-dehydrogenase	14	52.8	6.29	0.562	0.0022
W5HLM1	Uncharacterized protein	4	13.8	11.49	0.562	0.0035
A0A3B6FNB6	PALP domain-containing protein	4	58.2	7.02	0.568	0.0135
A0A3B5XVY7	Dihydrolipoyl dehydrogenase	15	59.1	6.73	0.569	0.0030
A0A3B5ZVU7	Uncharacterized protein	10	34.8	6.96	0.57	0.0050
A0A3B6MRT6	TPT domain-containing protein	2	42.6	10.15	0.572	0.0349
A0A3B6RFC6	O-phosphoserine phosphohydrolase	2	32.2	5.25	0.576	0.0091
A0A3B6JH94	Fumarate hydratase	18	53.2	7.61	0.578	0.0224
A0A3B6KHJ3	Protein kinase domain-containing protein	6	39.4	6.47	0.579	0.0061
A0A3B5YXX9	Uncharacterized protein	4	68.6	6.77	0.58	0.0080
W5GQY7	Ubiquitin thioesterase OTU1	6	23.2	5.17	0.581	0.0131
A0A3B6NR24	Isocitrate dehydrogenase [NAD] subunit, mitochondrial	10	43.8	6.73	0.582	0.0066
A0A3B5ZR06	Usp domain-containing protein	4	17.8	6.54	0.582	0.0037
A0A3B6KP84	Cytochrome b-c1 complex subunit 7	6	14.5	9.57	0.584	0.0070
A0A3B5Z0L3	PRA1 family protein	2	23.1	9.04	0.585	0.0458
A0A3B6TDM1	Ubiquitin-like modifier-activating enzyme 5	6	46.2	4.78	0.59	0.0179
A0A3B6KBK7	Nucleoside diphosphate kinase	2	24	9.32	0.59	0.0440
A0A3B6MVJ2	Transmembrane 9 superfamily member	11	73.3	7.21	0.591	0.0144
W5E6W1	Uncharacterized protein	6	15	10.48	0.593	0.0392
A0A3B6SJF6	Uncharacterized protein	2	19.3	9.61	0.593	0.0392
G0Z6F1	26S proteasome regulatory subunit RPN11	4	34.2	6.46	0.594	0.0101
A0A1D5THS1	Genome assembly, chromosome: II	4	13.8	11.49	0.594	0.0105
A0A3B6PSV9	Epimerase domain-containing protein	14	44.7	9.35	0.595	0.0076
U5HTD8	Ribosomal protein S20	7	14.1	9.44	0.595	0.0235
A0A3B6GXI9	Uncharacterized protein	4	48.8	6.87	0.598	0.0128
A0A3B5YZE8	Guanosine nucleotide diphosphate dissociation inhibitor	18	49.7	6.15	0.605	0.0171
A0A3B6IWB7	Phytocyanin domain-containing protein	4	21.1	6.51	0.606	0.0161
W5HTZ1	Uncharacterized protein	5	19.7	10.26	0.608	0.0255
A0A3B6RHV8	40S ribosomal protein S24	4	15.7	10.71	0.608	0.0149
A0A2X0S7E3	Prohibitin GN=CAMPLR22A2D_LOCUS3278	7	30.6	6.98	0.611	0.0208
A0A3B6AY90	Phytocyanin domain-containing protein	3	20.5	8.81	0.614	0.0269
A0A3B6LYD6	3Beta_HSD domain-containing protein	9	36.3	6.84	0.617	0.0259

A0A341W842	Plug_translocon domain-containing protein	6	52.5	8.98	0.624	0.0229
A0A1D5V328	Genome assembly, chromosome: II	5	18.1	4.41	0.626	0.0300
A0A3B6HS82	Uncharacterized protein	7	27.1	5.94	0.63	0.0500
A0A3B6FMI6	Glutaredoxin domain-containing protein	3	23.9	7.21	0.63	0.0432
A0A1D5XTG1	ER membrane protein complex subunit 4	3	19.2	9.04	0.631	0.0398
A0A3B6KCM1	Dihydrolipoamide acetyltransferase component of pyruvate dehydrogenase complex	9	47.4	7.94	0.635	0.0426
A0A3B6U6Q6	Dolichyl-diphosphooligosaccharide--protein glycosyltransferase subunit 1	6	52.5	7.69	0.635	0.0421
A7UME2	Xylanase inhibitor 725ACCN	7	41.2	7.52	0.645	0.0439
A0A3B5XX46	Peroxidase	8	37.6	5.05	1.41	0.0303
A0A3B6IUL0	Protein kinase domain-containing protein	3	91.1	7.17	1.467	0.0159
W5ACP2	BOWMAN_BIRK domain-containing protein	2	9.6	8.35	1.483	0.0201
A0A3B6QDR2	Uncharacterized protein	22	60.1	4.81	1.552	0.0463
A0A3B6PT24	Uncharacterized protein	6	81.6	7.36	1.584	0.0428
A0A3B6IRG1	Glucan endo-1,3-beta-D-glucosidase	2	48.9	6.32	1.592	0.0290
A0A3B6LSY3	Lipoxygenase	35	96.3	6.01	1.624	0.0011
A0A2X0S1X0	Genome assembly, chromosome: II	2	8.7	5.41	1.634	0.0458
A3RCW1	Translation initiation factor eIF5	5	49	5.71	1.652	0.0408
A0A3B5Y2A2	DNA replication licensing factor MCM2	11	98.7	5.99	1.744	0.0064
A0A3B6GRI2	NAD(P)H dehydrogenase (quinone)	3	22.5	6.3	1.782	0.0040
A0A3B6TMJ9	Protein kinase domain-containing protein	2	111.5	5.9	1.785	0.0047
A0A3B6QA61	TPR_REGION domain-containing protein	6	45.1	4.96	1.792	0.0037
W5CVU1	Uncharacterized protein	2	36.9	8	1.795	0.0049
A0A3B6DBW8	FAD-binding PCMH-type domain-containing protein	5	58.5	8.38	1.798	0.0045
Q7DMG9	Calmodulin	5	16.8	4.27	1.813	0.0000
A0A3B6GY97	Uncharacterized protein	2	39.2	5.88	1.817	0.0231
A0A3B6CJE1	Peroxidase	4	36.3	6.79	1.871	0.0010
A0A3B5YYP9	Uncharacterized protein	2	13.2	8.94	1.916	0.0014
Q56TP7	Beta-expansin TaEXPB5	2	29.8	8.51	2.044	0.0001
A0A3B5ZXA9	CASP-like protein	2	19.8	9.51	2.047	0.0312
A0A3B6N3E9	Acyl carrier protein	2	15	5.99	2.057	0.0166
A0A3B6NY49	Uncharacterized protein	5	57.2	9.26	2.121	0.0209
W5CY88	Glutaredoxin-dependent peroxiredoxin	6	17.3	5.31	2.139	0.0001
A0A3B6BXY2	Stress-response A/B barrel domain-containing protein	2	12.2	5.67	2.158	0.0166
A0A3B6RNB2	Uncharacterized protein	2	76.9	8.5	2.17	0.0214
A0A3B6KE54	Usp domain-containing protein	2	18.3	6.84	2.209	0.0439
A0A3B6PKH5	Glucose-6-phosphate 1-dehydrogenase	6	58.3	6.04	2.228	0.0369
A0A3B6C6N8	Myb_DNA-bind_3 domain-containing protein	2	39.9	7.11	2.255	0.0485
A0A3B6KJU3	TFIIB-type domain-containing protein	2	34.3	6.43	2.358	0.0431
A0A3B6I0G2	Adenylyl cyclase-associated protein	10	50.5	7.11	2.374	0.0317
A0A3B6JL36	SHSP domain-containing protein	2	24.1	5.4	2.456	0.0001
A0A3B6D9F6	L-lactate dehydrogenase	5	37.9	7.06	2.709	0.0056
A0A3B6TXL6	Ribosomal_L7Ae domain-containing protein	4	13.1	9.31	2.788	0.0055
A0A0C4BKA8	AAA domain-containing protein	15	46.7	8.98	3.022	0.0000
A0A3B6DK42	Uncharacterized protein	8	57.6	5.9	3.077	0.0001
A0A3B5Z0X4	Uncharacterized protein	2	45.5	6.61	3.146	0.0014

A0A3B6NRH4	PPR_long domain-containing protein	12	75.1	7.12	3.186	0.0047
A0A3B6QFP1	CN hydrolase domain-containing protein	2	36.2	5.66	3.243	0.0003
A0A3B6B068	Aldo_ket_red domain-containing protein	4	38	6.39	3.301	0.0026
A0A3B5ZT18	Uncharacterized protein	18	112.6	5.71	3.399	0.0000
A0A3B6TN49	Germin-like protein	2	25.5	8	3.509	0.0000
A0A3B6LI60	1-acylglycerol-3-phosphate O-acyltransferase	2	38.8	9.88	3.664	0.0000
A0A3B6MMS9	1-acylglycerol-3-phosphate O-acyltransferase	2	38.8	9.74	5.547	0.0000
A0A3B6AWI8	Uncharacterized protein	4	46.2	5.12	7.368	0.0000
A0A3B6D5D3	Stress-response A/B barrel domain-containing protein	2	12.2	5.67	7.885	0.0000
A0A3B6AX46	Succinate dehydrogenase [ubiquinone] flavoprotein subunit	13	68	6.68	10.736	0.0000
A0A3B6JFL4	Dirigent protein	4	19.9	7.15	10.82	0.0000
A0A3B6SCV1	Glycosyltransferase	2	52.1	5.43	27.191	0.0000
A0A3B6KTN2	Uncharacterized protein	9	35.4	5.63	100	0.0000
A0A3B5Y7S1	FAD-binding PCMH-type domain-containing protein	6	59.6	8.38	100	0.0000
A0A3B6LKE7	Uncharacterized protein	5	26.7	8.28	100	0.0000
A0A3B6RDW2	AB hydrolase-1 domain-containing protein	4	57.4	7.15	100	0.0000
A0A3B5YU68	CYTOSOL_AP domain-containing protein	2	55.4	6.16	100	0.0000
A0A3B6TJI0	Peroxidase	2	34.1	7.59	100	0.0000
A0A3B6B3Z5	Uncharacterized protein	2	93	6.23	100	0.0000

Abbreviation means as follows: Cov, coverage; MP, matched protein; MW, molecular weight; Cal, calculated.
Abundance Ratio: (Sample) / (Control) .
